# Dropout in supervised small-group exercise programs: a 7-year retrospective cohort study

**DOI:** 10.3389/fpubh.2026.1710202

**Published:** 2026-05-08

**Authors:** Carlos Eduardo Rosa da Silva, Wilian de Jesus Santana, Vinicius Morales, Luis Gustavo Pinto, Guilherme Leme, Antonio Roberto Doro, Leonardo Lima, Mary Elizabeth Jung, Aylton Figueira Junior

**Affiliations:** 1 GETAFIS – São Judas Tadeu University, São Paulo, SP, Brazil; 2 University of British Columbia – Okanagan Campus, Vancouver, BC, Canada

**Keywords:** exercise dropout, older people, personalized small-group training, supervised programs, survival analysis

## Abstract

**Introduction:**

Dropout to exercise programs remains a relevant challenge for physical education and public health professionals. Supervised small-group training is a promising strategy to enhance long-term adherence; however, evidence on its effectiveness in preventing dropout is still limited.

**Objective:**

To investigate participation duration and predictors of dropout in a supervised small-group training program using retrospective data from a personalized studio to analyze the mean length of participation and factors associated with dropout among adults.

**Methods:**

A retrospective cohort study was conducted with 587 participants enrolled between 2018 and 2024 at a training studio in São Paulo, Brazil. Variables analyzed included sex, physical activity level, body mass index (BMI), and reasons for initiating exercise. Kaplan–Meier survival analysis, Cox regression, and a decision-tree model (CHAID) were applied.

**Results:**

Mean participation was 13.8 ± 7.8 months, with a 12-month survival rate of 51.7%. Factors significantly associated with shorter participation were: male sex (HR = 0.614; *p* < 0.001), BMI ≥ 25 kg/m^2^ (HR = 1.220; *p* < 0.05), weight-loss as the main goal (HR = 0.639; p < 0.001). The decision tree identified male sex, weight-loss goals, and insufficient physical activity as the strongest predictors of dropout (*p* < 0.05).

**Conclusion:**

Dropout from supervised small-group training programs followed a progressive pattern, with nearly half of participants leaving within the first year. Male sex, overweight status, weight-loss goals complaints were the strongest predictors of early discontinuation, and the decision-tree analysis revealed that the combination of these factors further increased dropout risk.

## Introduction

Regular physical activity is widely recognized for its role in preventing and managing chronic non-communicable diseases, including cardiovascular disease, type 2 diabetes, and certain cancers (1–5). Moreover, active lifestyles contribute to improved mental health, reducing symptoms of depression and anxiety, and enhancing overall well-being (6–8). Physiological adaptations include improvements in cardiovascular function, glycemic control, body composition, and functional capacity (9–11).

Conversely, discontinuation of exercise can result in partial or complete loss of these adaptive responses (12). Previous studies (5, 12, 13) indicate that declines in cardiorespiratory fitness, muscular strength, and metabolic control may occur within weeks or months after detraining. In addition, body fat tends to increase, and susceptibility to chronic conditions such as hypertension, diabetes, and metabolic disorders rises, compromising the preventive and therapeutic benefits of exercise (13, 14).

Gyms can be considered a promising environment for promoting regular exercise because they provide structured routines, professional supervision, and opportunities for social interaction that encourage people to stay active (15, 16). These characteristics help increase confidence in performing exercises, support autonomy, and create meaningful social connections, which are key factors for long-term participation according to Self-Determination Theory (17). In addition, fitness centers offer safe and organized spaces with equipment that can be adjusted to individual needs and professionals who provide feedback and guidance. This combination allows progression, goal monitoring, and greater enjoyment, which contribute to making gyms a relevant and supportive setting for maintaining regular physical activity (18, 19).

Despite widespread knowledge of these benefits, maintaining exercise adherence over the long term remains challenging. Gyms, which are common venues for structured programs, often report dropout rates of 40–70% within the first 6 months (16, 20–24). This trend undermines the preventive potential of exercise and represents a significant barrier to sustained health promotion (20–22).

Small-group supervised training has emerged as a promising approach to promote long-term engagement in exercise programs. This model combines the benefits of individualized supervision with the motivational and social dynamics of a collective setting. According to Self-Determination Theory (17), feelings of relatedness, competence, and autonomy are central to sustaining motivation. Within small groups, participants experience greater social support, accountability, and shared goals, which foster a sense of belonging and increase intrinsic motivation to continue exercising (16, 18). From the perspective of Social Cognitive Theory (25), observing peers’ efforts and receiving feedback from professionals enhances self-efficacy and confidence in one’s ability to perform exercises correctly and safely. Furthermore, the group format facilitates regular attendance through interpersonal encouragement, promotes the development of technical skills through individualized feedback, and reduces injury risk through continuous supervision (19, 26). These mechanisms collectively contribute to greater enjoyment, consistency, and satisfaction with the exercise experience, making small-group supervision an effective strategy to reduce dropout and strengthen long-term adherence.

Nevertheless, few studies have examined the temporal dynamics of dropout in small-group settings, and even fewer have employed predictive models such as decision trees to identify hierarchical risk profiles. Thus, the present study aimed to analyze average participation duration and predictors of dropout in supervised small-group training programs. As a distinguishing feature, we employed Cox regression and decision-tree modeling using 7 years of retrospective data from a personalized training studio.

## Methods

This retrospective observational cohort study was conducted in a personalized training studio in São Paulo, Brazil. The facility (110 m^2^) was exclusively dedicated to exercise sessions, with a service model based on small groups (maximum of five participants per session). All exercise sessions were continuously supervised by two physical education professionals, each holding a bachelor’s degree in Physical Education and certification in strength and aerobic training. Both had more than 5 years of practical experience in individualized and group exercise prescription. Their professional background ensured that participants received evidence-based guidance, appropriate load progression, and continuous monitoring of exercise execution and safety throughout the sessions.

Records from participants who joined and discontinued the program between January 2018 and December 2024 were analyzed. Data were extracted from initial physical evaluation forms, allowing retrospective monitoring of adherence duration.

Sessions were pre-scheduled and consisted of three components: (i) warm-up with standardized stretches (5–10 min), (ii) resistance training (35–40 min), and (iii) aerobic exercise on treadmills or stationary bicycles (10–15 min). Prescriptions were individualized according to fitness level, characterizing a personalized small-group approach (12, 13). The studio enrolled an average of 12 ± 4.4 new participants per month in groups of 5 participants per session. The instructional style adopted by the professionals emphasized autonomy support, encouraging participants to actively engage in decision-making during sessions. Participants were guided to self-select exercise intensities during both aerobic and resistance training, within safe and individually appropriate ranges. This approach was particularly relevant given that the majority of participants were older adults, for whom fostering autonomy, perceived competence, and self-regulation is essential to promote motivation and long-term adherence.

### Participants

Eligible participants were adults (≥18 years) who had both entry and exit records in the program during the study period. Inclusion required complete data on physical evaluation and length of stay.

Exclusion criteria included: (i) incomplete entry/exit data, (ii) temporary interruptions with subsequent return (to avoid duplication and bias in survival analysis), and (iii) participants still active as of December 2024.

**Figure 1:**
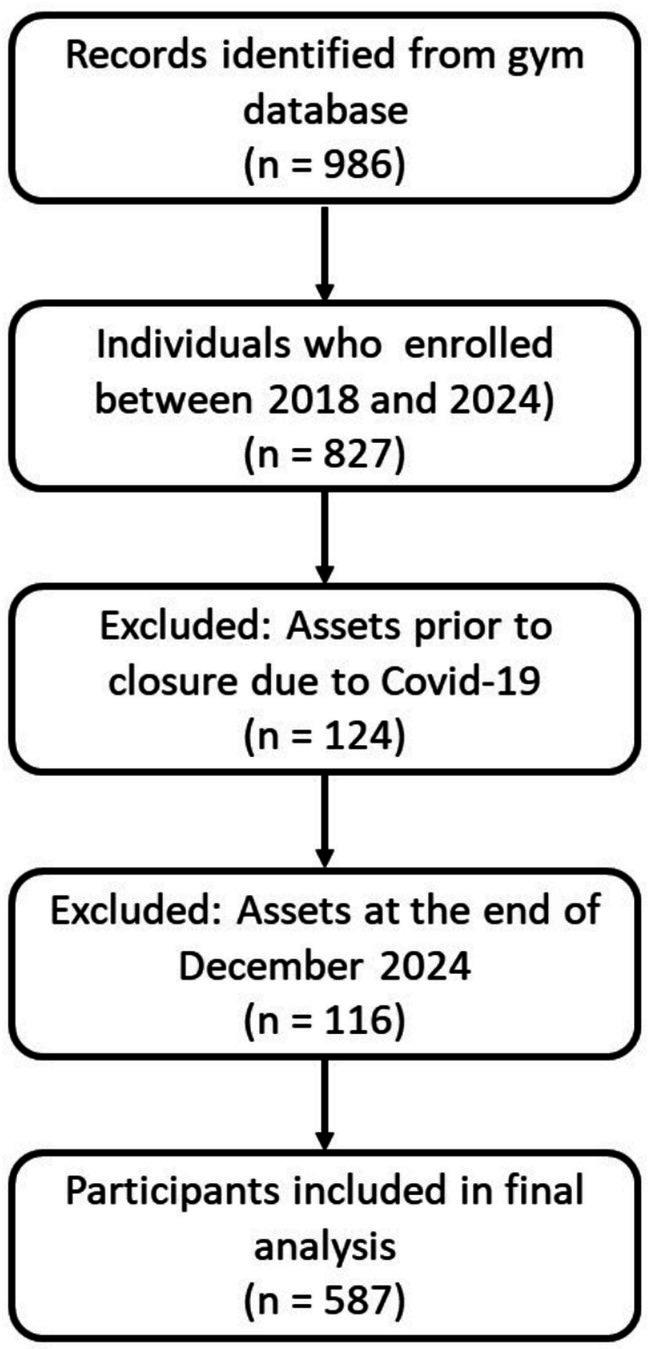
Flowchart for the selection of study participants.

A flowchart of participant eligibility and inclusion is shown in Figure 1.

Of the 986 records initially identified in the studio’s database, 827 individuals began the training program between 2018 and 2024. Among these, 124 participants who discontinued exclusively during the mandatory closure due to the COVID-19 pandemic (March 2020–July 2021) were excluded. Additionally, 116 participants were excluded because they were still active as of December 2024. In total, 587 participants met the inclusion criteria and were considered in the final analysis.

Exclusion of participants during the pandemic period was necessary, as interruption was not a voluntary decision but rather an external imposition, which could bias the accuracy of adherence analyses. Therefore, only periods of regular gym operation were considered, including participants who enrolled after reopening in August 2021.

Dropout monitoring was based on the dates of the first and last recorded sessions in the studio’s system. The date of the first session was considered the start of participation, while the date of the last session was considered program abandonment. Participants who did not return after their last recorded session were classified as inactive.

At baseline, anthropometric assessments were conducted, including height (cm) and body weight (kg), BMI (kg/m^2^). In addition, the short version of the International Physical Activity Questionnaire (IPAQ) (27) was administered to classify participants by physical activity level. Those reporting ≥150 min of weekly activity were considered “physically active,” while those with <150 min/week were classified as “insufficiently active.”

All assessments and records used in this study were part of the studio’s standard operational routine and not implemented specifically for research purposes. As this was a retrospective analysis, data were obtained from the facility’s digital management system, which routinely stored participants’ entry evaluations, training logs, and attendance information. No procedures were altered or introduced for the purpose of the study, and the researchers accessed the existing database only after program completion to conduct the analyses.

The evaluation forms also contained a specific field for participants to indicate their primary goals for joining the program. The most frequent responses were grouped into categories: weight loss, physical conditioning, health improvement, muscle hypertrophy, well-being, pain treatment, leisure, and posture correction. These variables were used for sample characterization and analysis of factors associated with program adherence. This measure was not based on a standardized or validated instrument. As part of the studio’s regular intake procedure, all new participants completed an initial physical evaluation that included an open-ended question asking for their main reason for seeking regular exercise practice. Responses were recorded in the participants’ digital files within the management system. During data extraction for this study, these records were reviewed, and the reported reasons were categorized into common themes. This study was approved by the Research Ethics Committee São Judas Tadeu University (São Paulo, Brazil), protocol no. 4,583,831. All procedures complied with ethical principles of confidentiality and anonymity, in accordance with the Declaration of Helsinki.

### Statistical analysis

Statistical analyses were performed using SPSS software, version 28.0 (IBM Corp., Chicago, IL, United States). Descriptive analyses of continuous variables were expressed as means and standard deviations. The dependent variable was the length of stay (months) in the supervised exercise program. Cox proportional hazards regression was applied to identify factors associated with shorter participation. Independent variables included sex, age, physical activity level, and reported initial goals (physical conditioning, weight loss, leisure, well-being, hypertrophy, health, pain treatment, and posture improvement). Statistical significance was set at *p* < 0.05. The selection of independent variables was based on previous empirical evidence and theoretical relevance to exercise behavior and adherence. Sex and age were included because demographic characteristics have consistently been associated with participation patterns and dropout risk in exercise programs (23, 26). Baseline physical activity level was considered an indicator of behavioral readiness and prior habit strength, factors that influence the likelihood of maintaining regular participation over time (18). Body mass index (BMI) was included due to its known relationship with exercise motivation and perceived barriers, particularly among overweight and individuals with obesity (24). Finally, participants’ initial goals for joining the program such as weight loss, health improvement, pain reduction, and well-being were analyzed because motivational orientation and goal content are strong determinants of adherence according to the Self-Determination Theory (17) and have been empirically linked to exercise persistence in supervised and unsupervised settings (16, 21).

To describe dropout dynamics over time, Kaplan–Meier survival analysis was conducted, estimating mean, median, and 12-month retention. Decision-tree analysis (CHAID method) was used to identify the main hierarchical predictors of adherence. Variables included sociodemographic factors (sex, age), behavioral factors (physical activity level), and motivational factors (weight loss, health, pain treatment, hypertrophy, fitness, well-being, leisure, aesthetics, posture). The model was parameterized with a maximum depth of three levels, allowing up to three successive splits from the root node.

The chi-square test was adopted as the splitting criterion, with Bonferroni adjustment to control type I error. Minimum sample sizes were set at 50 cases per internal node and 30 cases per terminal node to ensure robustness. Automatic partitioning was applied to maximize associations between predictors and program adherence. Cross-validation was not applied, as the objective was to generate an interpretative, descriptive model exploring hierarchical profiles within the sample. The resulting tree illustrated the main predictors of adherence, with nodes reflecting different mean lengths of stay based on variable combinations with the greatest discriminatory power for dropout.

## Results

Table 1 presents the Cox regression results for factors associated with dropout from supervised small-group exercise programs.

**Table 1:** The Cox regression results for factors associated with dropout from supervised small-group exercise programs.

Variables	*N*	%	B	IF	Wald	df	*p*-value	Exp (B)	IC 95%
Age									
Over 63 years old	304	51.8%	0.11	0.08	1.66	1	0.19	1.11	0.94–1.31
< 63 years old	283	48.2%							
≤ 52 years old	148	25.2%							
53–63 years old	150	25.6%							
64–74 years old	156	26.6%							
≥ 75 years old	133	22.7%							
Sex									
Male	158	26.9%	−0.48	0.09	24.51	1	0	0.61	0.50–0.74
Female	429	73.1%							
BMI									
Normal	144	24.5%							
Above	443	75.5%	0,19	0.10	4.11	1	0.04	1.22	1–1.47
Physical activity level									
Active	116	19.8%							
Insufficiently active	471	80.2%	−0.46	0.09	0.18	1	0.67	0.95	0.77–1.18
Reason: Cond. Physical	*N*	%							
Yes	180	30.7%	0.94	0.09	1.10	1	0.29	1.09	0.92–1.31
No	407	69.3%							
Reason: Weight loss									
Yes	469	79.9%	−0.44	0.10	17.14	1	0	0.63	0.52–0.78
No	118	20.1%							
Reason: Leisure									
Yes	26	4.4%	0.07	0.20	0.13	1	0.71	1.07	0.72–1.61
No	561	95.6%							
Reason: Wellness									
Yes	57	9.7%	−0.04	0.14	0.09	1	0.76	0.95	0.72–1.27
No	530	90.3%							
Reason: Hypertrophy									
Yes	403	68.7%	−0.11	0.09	1.73	1	0.18	0.88	0.74–1.06
No	184	31.3%							
Reason: Improved health									
Yes	468	79.7%	0.05	0.10	0.26	1	0.60	1.05	0.86–1.3
No	119	20.3%							
Reason: Pain treatment									
Yes	22	3.7%	−0.56	0.22	6.42	1	0.11	0.56	0.36–0.89
No	565	96.3%							
Reason: Improved posture									
Yes	29	4.9%	0.12	0.19	0.39	1	0.52	1.12	0.77–1.65
No	558	95.1%							
Omnibus test		−2 Log	*χ* ^2^	df	*p*-value				
		6,398,21	46,726	12	0				

B, regression coefficient estimated by the Cox model; IF, standard error of the coefficient; Wald, Wald test statistics; df, degrees of freedom; *p*, level of statistical significance of the association; and Exp(B), hazard ratio, expressing the effect of the variable on the time to the event (dropout). The 2 Log statistic, negative likelihood of the model; *χ*^2^, chi-square Omnibus test, used to assess the overall significance of the model; df, degrees of freedom of the model in the global test, and *p*-value, significance.

For age, participants were divided into two groups (≤63 and >63 years). The cutoff point of 63 years corresponded to the sample median, allowing balanced distribution between categories for regression analysis. This choice was made for analytic transparency and ease of interpretation in Kaplan–Meier plots and in the decision-tree output, and it avoids undue influence of extreme values. The 63-year threshold is not intended to represent a clinical cutoff or a policy boundary; it simply reflects the empirical distribution of our cohort. We recognize that dichotomization reduces information relative to treating age as continuous and we interpret age effects cautiously in light of this limitation.

The age of participants ranged from 40 to 85 years, with a median of 63 years and an interquartile range of 52–74 years. When classified into age groups, 0% were younger than 25 years, 0% were between 25 and 44 years, 46.8% were between 45 and 64 years, and 53.2% were 65 years or older. This distribution indicates that the sample was predominantly composed of middle-aged and older adults, reflecting the population typically engaged in supervised exercise programs in small-group formats.

The Cox regression analysis revealed significant associations between specific factors and the risk of dropout from the supervised small-group training program. Male participants demonstrated a shorter duration of adherence, with a hazard ratio (HR) of (*p* < 0.001), indicating a higher likelihood of dropout compared with females. Likewise, reporting weight loss as the primary training goal was associated with increased dropout risk (*p* < 0.001).

BMI ≥ 25 kg/m^2^ also emerged as a significant factor (*p* < 0.05), suggesting that individuals with overweight or obesity were at higher risk of dropout. In contrast, age, baseline physical activity level, and other reported goals (e.g., hypertrophy, well-being, health improvement) were not significantly associated with program retention. The overall model fit was statistically significant (*p* < 0.001), confirming that the set of independent variables contributed meaningfully to explaining dropout risk.

Kaplan–Meier survival analysis was performed to estimate the mean and median duration of program participation across the entire follow-up period. Because this analysis was conducted descriptively and without subgroup comparisons, neither the log-rank test nor *p*-values were reported. Instead, the results represent the temporal distribution of adherence across the total sample, allowing characterization of participation dynamics without statistical inference between groups.

Table 2 summarizes the Kaplan–Meier estimates of program retention.

**Table 2:** Kaplan–Meier estimates of total length of stay in the exercise program.

Statistics	Estimate	EP	95% CI
Average time (months)	13.8	0.32	13.15–14.42
Median time (months)	13	0.63	11.77–14.23

Estimates obtained through Kaplan–Meier survival analysis. EP, standard error of the mean. 95% CI, 95% confidence interval for the mean; *p*-value, level of statistical significance.

The results presented in Table 2 indicate that the mean length of stay in the exercise program. To allow comparison with previous studies (16, 23, 24), we conducted an additional analysis truncating follow-up at 12 months, examining the association between sex and program adherence.

Inclusion of sex in the Kaplan–Meier analysis was warranted by the Cox regression findings, which identified male sex as a significant predictor of shorter participation. Male presented a 39% higher risk of dropout compared with women (*p* < 0.001). Kaplan–Meier survival curves stratified by sex illustrate the temporal adherence patterns, highlighting the greater vulnerability of men to early discontinuation and providing insight into subgroup-specific dropout dynamics.

**Figure 2:**
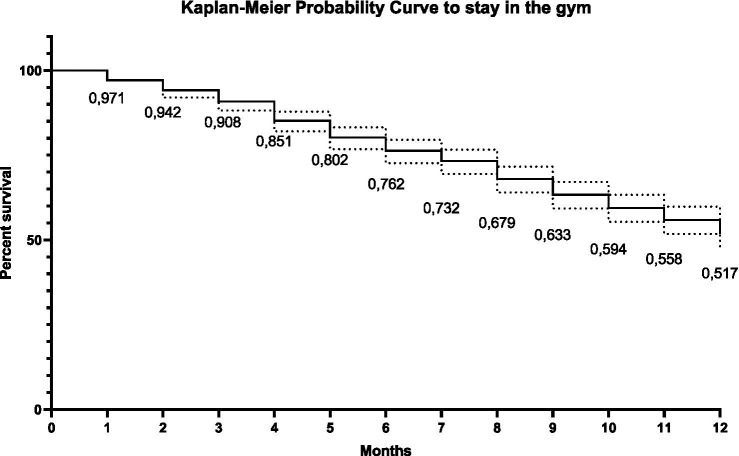
Kaplan–Meier curve representing the cumulative probability of participants remaining in the exercise program over 12 months. The values below the curve indicate the proportions of permanence (survival) in months.

The survival analysis demonstrated that Table 3. The mean duration of participation in the training program was significantly shorter among men (mean = 8.54 months) compared with women (mean = 9.77 months). The overall mean duration was 9.44 months. The log-rank test confirmed a statistically significant difference between sexes (*p* < 0.001). These results corroborate the Cox regression findings, which identified male sex as a significant predictor of increased dropout risk (Figure 2).

**Table 3:** Kaplan–Meier estimates of total length of stay in the exercise program over a 12-month period.

Sex	Average time (months)	EP	95% CI
Male	8.54	0,27	7.99–9.08
Female	9,77	0,16	9.44–10.09
Total (overall)	9.44	0,14	9.15–9.72

	*χ* ^2^	Df	*p*-value
Log-rank (Mantel-Cox):	32,68	1	0

*χ*^2^, statistics of the Log-Rank test; df, degrees of freedom; EP, standard error of the mean; 95% CI, 95% confidence interval for the mean; *p*-value, level of statistical significance.

**Figure 3:**
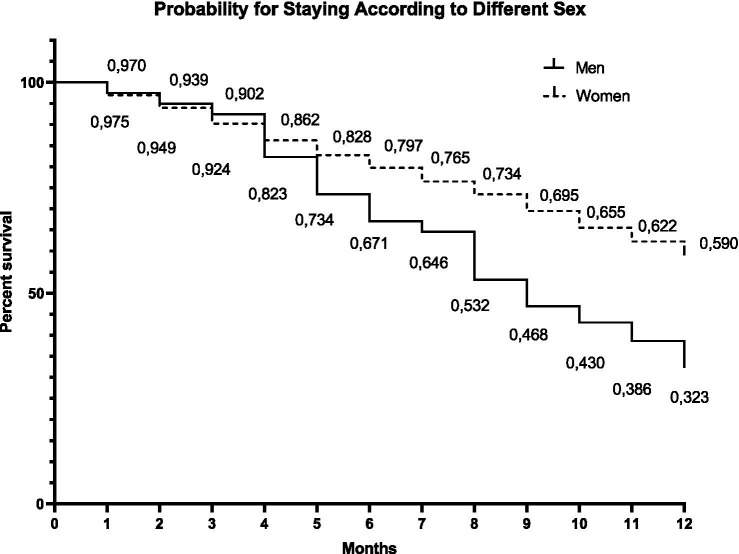
Kaplan–Meier curve representing the cumulative probability of permanence of participants in the exercise program over 12 months compared between sexes. The values below the curve indicate the proportions of permanence (survival) in months.

The survival curve demonstrated a progressive decline in program participation over the 12-month follow-up. In the first month, approximately 97% of participants remained enrolled; however, this proportion decreased steadily, reaching 51.7% at the end of 12 months (Figure 3).

The survival analysis stratified by sex revealed significant differences in program participation between men and women. The mean participation time for men was 8.54 months, compared with 9.77 months for women, with a statistically significant difference (*p* < 0.001). The Kaplan–Meier curves demonstrated a steeper decline in survival probability among men across follow-up, reinforcing the Cox regression findings that identified male sex as a significant predictor of dropout risk.

**Figure 4:**
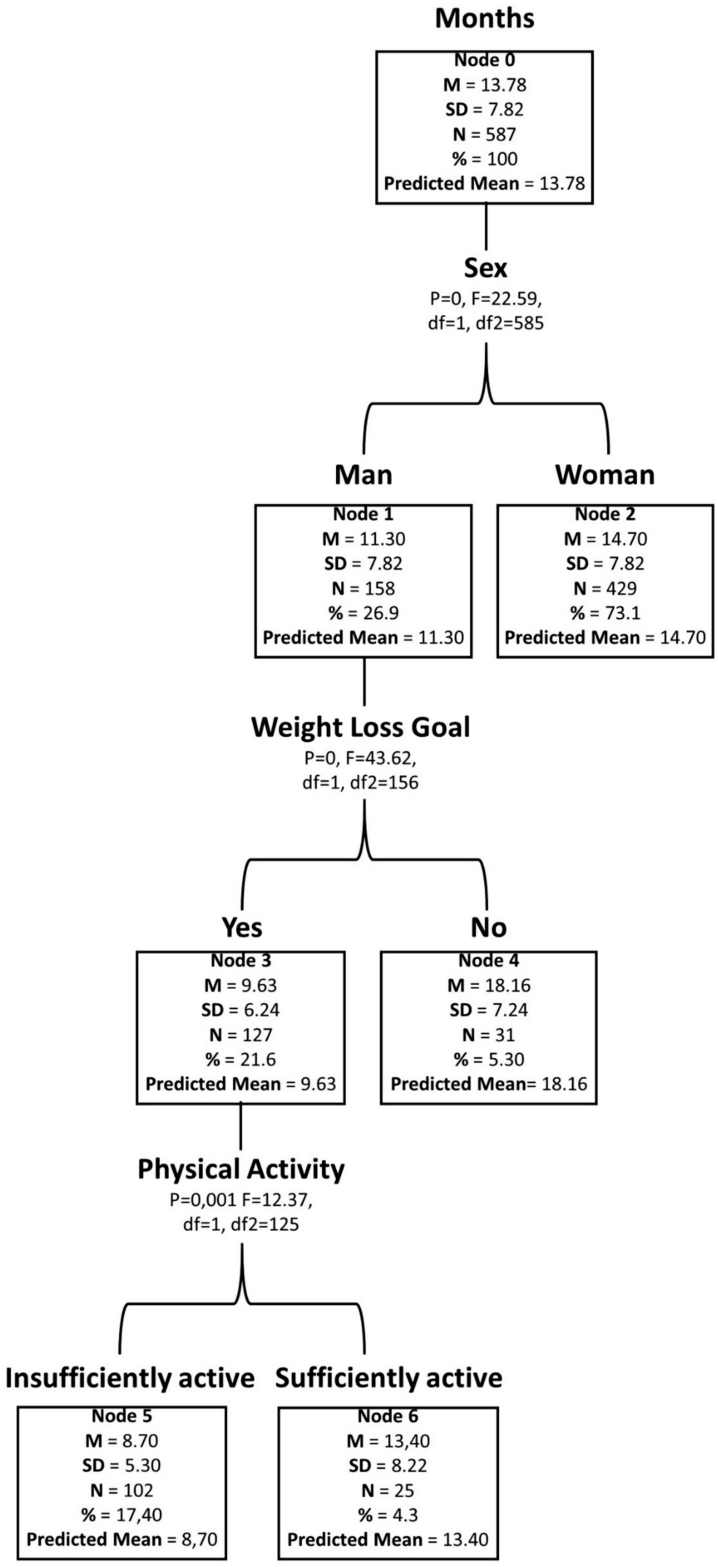
Decision-tree model generated using length of stay (months) in the exercise program as the dependent variable. Average, average length of stay in the program (in months); Deviation Pad., standard deviation of the mean; *N*, number of participants in the group; %, percentage in relation to the total sample; predicted, average value predicted by the model; *p*-value, adjusted level of statistical significance of the discriminant factor; F, statistics of the *F*-test for knot division; df, degrees of freedom.

The decision tree presented in Figure 4 illustrates the main hierarchical predictors associated with program participation. The initial node (Node 0) represented the overall mean length of stay, which was 13.78 ± 7.82 months. The first discriminating factor was sex: men (Node 1) showed a mean participation time of less than 11.30 months, compared with women (Node 2), who remained on average 14.70 months (*p* < 0.001; *F* = 22.59).

Among men, the second relevant factor was the weight-loss goal. Those who entered the program with this primary motivation (Node 3) had the shortest average participation time (9.63 months; *p* < 0.001; *F* = 43.62), whereas those without this goal (Node 4) stayed significantly longer, with a mean of 18.16 months (*p* < 0.05; *F* = 12.37). Furthermore, among men with weight loss as their main goal, baseline physical activity level emerged as the strongest discriminator: insufficiently active individuals (Node 5) had the shortest participation across the entire model (8.7 months), whereas sufficiently active individuals (Node 6) remained for an average of 13.4 months.

## Discussion

This study investigated participation duration and predictors of dropout in a supervised small-group training program, using retrospective data from a personalized studio. The mean participation time was 13.8 months (SE = 0.32; 95% CI: 13.15–14.42), with a median of 13 months (SE = 0.63; 95% CI: 11.77–14.23). These values reflect substantially higher adherence than the average reported in conventional gyms, which typically ranges from 3 to 6 months (15, 20–22, 24, 26, 28, 29).

An important distinguishing feature of this study is the age profile of participants, composed primarily of older adults and older adults individuals (M = 62.9 ± 12.9 years). This contrasts with previous investigations (21, 23, 24, 29) that focused on younger and middle-aged populations, thus broadening understanding of adherence in older populations. The sample in this study consisted mainly of middle-aged and older adults, with more than half (53%) aged 65 years or older. This age profile is particularly relevant, as older adults tend to face greater physical, motivational, and logistical barriers to maintaining regular exercise participation (15). The predominance of this age group provides valuable insight into adherence patterns within a population that often requires closer supervision and individualized support. Furthermore, the inclusion of participants across a wide age range (40–85 years) allowed the analysis of adherence behaviors representative of both pre-retirement and post-retirement stages of life, where exercise motivation and perceived benefits may differ substantially.

Our findings are consistent with those of Sperandei et al. (23), who identified a dropout risk profile characterized by men with elevated BMI, low prior physical activity, and extrinsic goals such as weight loss. Similarly, our analysis revealed that men with overweight or with obesity and weight loss as their main goal exhibited shorter adherence. However, while Sperandei et al. (23) reported extremely high dropout, with only 3.7% of participants active after 12 months, the present small-group supervised training model demonstrated markedly higher retention, with 51.7% remaining at 12 months. This difference may be attributable to interpersonal support, social bonding (17, 30), and continuous supervision factors likely acting as protective elements against early dropout.

The observed gender differences in adherence are noteworthy and may reflect distinct motivational and behavioral patterns between men and women. Previous studies have shown that women often report aesthetic or health-related reasons, such as weight loss or body composition improvement, as primary motives for exercise initiation, while men tend to emphasize performance, strength, or competition-oriented goals (15, 31). These motivational differences may influence the type of commitment established with exercise, as extrinsically driven goals such as appearance or weight control are generally associated with lower persistence when outcomes are not immediately achieved (17, 18).

Additionally, social and cultural expectations may contribute to these patterns. Women often perceive exercise environments as spaces for social connection and emotional support, whereas men may prioritize autonomy and measurable progress (32). In the context of supervised small-group training, these dynamics may play a decisive role: the social support, feedback, and sense of belonging offered by this model appear to favor female participation, while men might disengage earlier when their performance-related goals or competitive motivations are not met. This interpretation aligns with evidence that intrinsic motivation, enjoyment, and relatedness predict sustained adherence, whereas external pressures or outcome-focused motives are linked to early dropout (16, 26).

The expertise and engagement of personal trainers appear to be decisive factors in reducing dropout from supervised exercise programs. When professionals adopt an autonomy-supportive and socially engaging style, they help participants feel competent, confident, and connected to the training process. Providing constructive feedback, correcting technique, and encouraging self-regulation foster greater satisfaction and a sense of progress, which are essential for sustaining motivation over time. Such behaviors are consistent with the principles of the Self-Determination Theory and have been associated with higher adherence and lower attrition rates in supervised exercise settings (16, 18, 33–35).

Cox regression confirmed that male sex (*p* < 0.001), high BMI (*p* < 0.05) and weight-loss goal (*p* < 0.001), were significant predictors of shorter participation. The decision tree further refined these associations, showing that the combined presence of male sex, weight-loss goals, and insufficient physical activity was strongly predictive of dropout.

Retention in this study was notably higher than in conventional gyms: Sperandei et al. (23) observed only 3.7% at 12 months, Gjestvang et al. (21) reported 37%, and Faro et al. (24) found that participants in High-Intensity Functional Training (HIFT) programs remained an average of just 90 days, with only 12.8% adherence after 1 year. In contrast, our program achieved 51.7% survival at 12 months, with an average participation of 13.8 months. These findings suggest that supervised small-group training, combining personalization, continuous professional oversight, and social support (17, 28, 30, 36), offers a substantially more effective model for retention.

In the present study, adherence was assessed based on the total duration of participation in the supervised small-group program, as recorded in the studio’s digital attendance system. Because this was a retrospective design, continuous training behavior throughout the participation period could not be objectively verified. Therefore, the analyses considered the time elapsed between the first and last recorded sessions as a proxy for adherence, assuming that participants who remained enrolled were exercising regularly within that period. Although this method does not capture fluctuations in attendance or temporary interruptions, it reflects the real-world dynamics of participation in private fitness settings, where payment and scheduling are typically linked to ongoing attendance. Future studies employing longitudinal follow-up or repeated attendance tracking similar to the approach used by Gjestvang et al. (21) would provide a more detailed understanding of how regularity and consistency evolve over time. Thus, the present findings should be interpreted as indicators of program retention rather than continuous weekly adherence.

The reasons for joining the exercise program help to clarify the dynamics of dropout. Although goals such as health improvement and physical conditioning are commonly reported as initial motives for starting a training routine, our findings indicate that weight loss, particularly when associated with male sex and higher BMI, was a strong determinant of early dropout (23, 26). It is important to emphasize that these motives reflect the reasons participants gave for initiating exercise, not the type or regulation of motivation itself. According to the Self-Determination Theory (17), the quality of motivation depends on the degree of internalization of these motives that is, whether individuals engage in exercise because they value it and find personal meaning (autonomous regulation) or because of external pressures and expectations (controlled regulation). Thus, a weight loss goal may not necessarily indicate extrinsic motivation if it is pursued for self-endorsed reasons such as health or self-care. However, when weight loss is driven primarily by external appearance related pressures, it tends to produce less stable engagement and higher dropout risk (18, 35). A more nuanced understanding of these distinctions suggests that the content of the initial motive is less predictive of adherence than the degree to which it is autonomously regulated over time.

The small-group training format itself may explain part of the higher adherence. Training in groups of three to five participants, under continuous professional supervision, combines individualized prescription with the motivational benefits of a collective environment (28, 36, 37). Prior research has highlighted those perceptions of professional support and a sense of belonging are key behavioral determinants for sustained physical activity (17, 19, 30). In this study, the interpersonal bonds fostered by the small-group setting likely contributed to the significantly longer adherence compared to traditional gyms.

Nevertheless, the dropout curve showed a continuous decline without stabilization, similar to patterns described in other studies (21, 23, 24). Nearly half of participants discontinued before 12 months, underscoring the importance of early interventions, particularly in the first 3 months a critical period for habit consolidation. Social relationships between trainers and participants appear to play a decisive role in mitigating this attrition.

## Limitations

This study has limitations. Its retrospective observational design and convenience sample of adults and older adults restrict generalizability to other settings such as conventional gyms, public health programs, or younger populations. Data were derived from enrollment records and did not include psychosocial constructs (e.g., social support perception, self-efficacy, satisfaction, barriers). Moreover, dropout was defined solely as program discontinuation, without distinguishing between voluntary (e.g., dissatisfaction, lack of results) and external causes (e.g., relocation, health issues, financial barriers). Consequently, results should be interpreted cautiously, and randomized controlled trials are needed to isolate the effects of social support, supervision, and personalization.

## Practical implications

Despite limitations, the findings have meaningful implications for the fitness market, public health, and clinical settings. For gyms, adopting supervised small-group models may substantially reduce dropout, enhance member retention, and strengthen client loyalty. Physical education professionals must be prepared not only to prescribe exercise but also to provide motivational support and foster social connectedness. For public policy, incorporating small-group supervised programs may improve adherence in high-risk populations such as individuals with overweight, individuals with obesity, or low-autonomy individuals. Clinically, rehabilitation centers and health promotion programs could adopt this model to ensure greater adherence, thereby improving long-term physical and mental health outcomes.

Additionally, it is important to acknowledge the potential influence of socioeconomic factors on the outcomes. Individuals who remained in the program for longer periods may have had more disposable income or greater access to health-promoting resources, which could limit the generalizability of the findings to broader or more vulnerable populations. This consideration highlights the need for future interventions and public initiatives to explore ways of making such supervised programs accessible and feasible across diverse socioeconomic backgrounds.

## Conclusion

This study examined participation duration and predictors of dropout in a supervised small-group exercise program using 7 years of real-world data. The findings revealed an average participation of 13.8 months and a 12-month retention rate of 51.7%, indicating that adherence gradually declined over time. Cox regression and decision-tree analyses consistently identified male sex, overweight status, weight-loss goals, and pain as the strongest predictors of early dropout. The combination of these factors characterized the most vulnerable profile, with significantly shorter participation duration.

These results directly answer the study question by showing that dropout in supervised small-group programs follows identifiable and non-random patterns influenced by biological and motivational characteristics. The findings highlight those men with excess body weight who begin training primarily for weight-loss or pain relief are at higher risk of discontinuation. Conversely, the small-group format itself, characterized by professional supervision, social support, and individualized feedback, may contribute to the higher overall retention observed compared with conventional gym settings.

Future research should include longitudinal tracking of attendance and psychosocial measures to better understand how motivational regulation and social interaction influence adherence trajectories in supervised exercise programs.

## Data Availability

The datasets presented in this study can be found in online repositories. The names of the repository/repositories and accession number(s) can be found in the article/Supplementary material.
